# Camelliasaponin B1, a Saponin from *Camellia oleifera* Seed, Protects Against Oxidative Stress and Is Associated with Reduced BNIP3/NIX-LC3B Expression in PC12 Cells

**DOI:** 10.3390/antiox15070824

**Published:** 2026-06-30

**Authors:** Xiaoqing Feng, Xiao Zhou, Shushan Jia, Jingzhen Chen, Peiwang Li, Yan Yang, Wei Wu, Lijuan Jiang, Wenbin Zeng, Changzhu Li, Qiang Liu, Yunzhu Chen

**Affiliations:** 1 College of Life Science and Technology, Central South University of Forestry and Technology, 498 South Shaoshan Road, Changsha 410004, China; 2 State Key Laboratory of Woody Oil Resources Utilization, Hunan Academy of Forestry, Changsha 410004, China; 3 Qingdao Research Academy of Environmental Sciences, Qingdao 266000, China

**Keywords:** camelliasaponin B1, oxidative stress, mitophagy, BNIP3/NIX, mitochondrial function, neuroprotection

## Abstract

Camelliasaponins, bioactive constituents abundant in the by-products of *Camellia oleifera* oil production, exhibit diverse biological activities. However, their potential in regulating neuroprotective mitophagy remains largely unexplored. This study identifies camelliasaponin B1 (CSB1) as an abundant component in *C. oleifera* seeds and investigates its cytoprotective mechanisms against oxidative stress. Using an in vitro model of H_2_O_2_-induced oxidative damage in PC12 cells, we found that CSB1 pretreatment significantly alleviated oxidative stress, as evidenced by reduced reactive oxygen species (ROS) accumulation and enhanced antioxidant enzyme activities (SOD, CAT, GSH-Px). CSB1 also preserved mitochondrial function, restoring membrane potential (ΔΨm), ultrastructure, and respiratory capacity. Mechanistically, CSB1 reduces the expression of BNIP3/NIX-LC3B pathway-related proteins, suggesting a modulatory effect on mitophagy, as supported by transcriptomic analysis, Western blotting, and immunofluorescence. Molecular docking computationally predicted potential interactions between CSB1 and BNIP3/NIX proteins, which require experimental validation. Collectively, these findings suggest that CSB1 acts as a cytoprotective agent that enhances antioxidant defenses, safeguards mitochondrial integrity, and is associated with reduced BNIP3/NIX-LC3B expression and co-localization, offering a potential molecular basis for its development as a neuroprotective agent targeting oxidative stress-related mitochondrial dysfunction.

## 1. Introduction

*Camellia oleifera* Abel. (*C. oleifera*) is a key woody oil species, primarily valued for the production of high-quality edible oil (i.e., camellia oil) [1]. The oil extraction process generates a considerable amount of by-product, specifically camellia seed cake. China’s annual output of camellia seed cake reaches 1.97 million tons. Currently, this by-product is mostly discarded without being exploited for high-value applications [2,3]. Camellia seed cake is rich in diverse bioactive compounds. Among these bioactive compounds, saponins have garnered significant attention due to their prominent biological activities, such as anticancer and antioxidant effects [4,5]. Among the various saponin constituents, Camelliasaponin B1 (CSB1), a characteristic bioactive monomer derived from camellia seed cake saponins, has a well-defined structure and notable biological activity and exhibits considerable antioxidant potential [6,7]. However, its specific biological functions, particularly the molecular mechanisms underlying its regulation of organelle functions, remain incompletely elucidated.

Neurodegenerative diseases, such as Alzheimer’s disease (AD) and Parkinson’s disease (PD), exhibit a continuous global increase in incidence alongside the aging population, posing an increasingly severe public health challenge [8,9]. Extensive studies have indicated that oxidative stress acts as a central pathological mechanism driving neuronal damage and apoptosis [10,11]. To simulate neuronal oxidative damage in vitro, hydrogen peroxide (H_2_O_2_) is widely used in various oxidative stress models, as it directly induces massive generation of reactive oxygen species (ROS) and subsequently leads to mitochondrial dysfunction (a key contributor to neuronal impairment) [12,13].

Mitochondria, serving as the cellular powerhouses and one of the primary sources of ROS production, are critical targets of oxidative stress-induced damage [14,15]. Sustained oxidative stress can disrupt mitochondrial membrane potential (MMP), impair oxidative phosphorylation (OXPHOS) activity, and trigger the mitochondrial apoptotic pathway [16,17]. For mitochondrial homeostasis, recent studies have revealed that mitophagy plays a dual role in the interplay between oxidative stress and neuronal damage: moderate mitophagy acts as an essential mechanism to clear damaged mitochondria and maintain cellular homeostasis [18]. However, excessive or dysregulated mitophagy can result in the elimination of functionally intact mitochondria, ultimately leading to energy depletion and cell death [19,20]. In the context of neuronal protection, among various mitophagic pathways, those mediated by mitochondrial receptor proteins (e.g., BNIP3, NIX, and FUNDC1) are particularly critical in regulating neuronal cell damage [21,22,23]. Therefore, targeting the axis of oxidative stress–mitochondrial dysfunction–excessive mitophagy has emerged as a pivotal strategy for developing neuroprotective interventions.

Therefore, this study aimed to investigate the cytoprotective effects of CSB1 against oxidative stress, with a focus on its role in regulating mitochondrial quality control via mitophagy. Using transcriptomic analysis, molecular docking, and functional assays, we sought to elucidate whether CSB1 protects PC12 cells from H_2_O_2_-induced oxidative damage by modulating the BNIP3/NIX-LC3B pathway. Elucidating this mechanism will not only provide novel insights into the compound’s antioxidant properties but also support the high-value utilization of this underutilized agricultural by-product.

## 2. Materials and Methods

### 2.1. Materials and Reagents

PC12 cells (a rat adrenal pheochromocytoma cell line) were acquired from the Beijing Beina Chuanglian Biotechnology Research Institute (Suzhou, China). CSB1 was provided by the Hunan Academy of Forestry (Changsha, China). H_2_O_2_ (30% *w*/*w*; No. H433856-500 mL) was acquired from Aladdin Biochemical Technology Co., Ltd. (Shanghai, China). High-glucose DMEM (No. PM150210), fetal bovine serum (No. 164210), 0.25% trypsin–EDTA (No. PB180225), phosphate-buffered saline (PBS) (No. PB180327) and 1% penicillin–streptomycin solution (No. PB180120) were purchased from Procell Life Science&Technology Co,. Ltd. (Wuhan, China). Reactive oxygen species (ROS) assay kits (No. CA1410) were purchased from Solarbio Science & Technology Co., Ltd. (Beijing, China). Superoxide dismutase (SOD) (No. A001-3), glutathione peroxidase (GSH-Px) (No. A005-1), catalase (CAT) (No. A007-1-1), and malondialdehyde (MDA) (No. A003-1) assay kits were purchased from Jiancheng Biochemical (Nanjing, China). A mitochondrial calcium assay kit (No. S1062S) and mitochondrial membrane potential and apoptosis detection kit (No. C1071S) were purchased from Beyotime Biotechnology (Shanghai, China). The CCK-8 assay kit (No. C6005) and the total RNA extraction kit (No. M5102) were sourced from NCM Biotech (Suzhou, China). A PrimeScript™ RT reagent kit (No. RR047A) and TB green premix ex Taq™ II (No. RR820Q) were purchased from Takara Bio Inc. (Dalian, China). Primers were synthesized by Tsingke Biotechnology Co., Ltd. (Beijing, China). Antibodies against β-actin (No. YM3028), BCL2 (No. YM3041), BAX (No. YT0455), BNIP3 (No. YM8655), and LC3B (No. YM8147) were obtained from ImmunoWay Biotechnology Company (Plano, Texas, USA). Antibodies against NIX (No. 68118-1-Ig) were obtained from Proteintech (Wuhan, China). All other reagents were of analytical grade and were purchased locally in China.

### 2.2. Extraction and Quantification of CSB1

For quantitative analysis, a Waters e2695 HPLC system equipped with an ELSD6000 evaporative light-scattering detector (Waters Corporation, Milford, MA, USA) (drift tube temperature 105 °C, nebulizer gas pressure 25 psi, and nitrogen flow rate 2.7 L/min) and a Zhongpulan XD-C18 column (4.6 × 250 mm, 5 μm) was used. The analysis was conducted at 35 °C with a flow rate of 1.0 mL/min for 60 min. The mobile phase consisted of acetonitrile (A) and 1% acetic acid aqueous solution (B), with the following gradient program: 0 min (36% A) → 20 min (46% A) → 25 min (90% A) → 35 min (90% A) → 36 min (36% A) → 48 min (36% A). An injection volume of 10 µL was used, and the target compound was identified by comparing its retention time with that of a CSB1 reference standard.

For tissue-specific distribution analysis, different tissues of *C. oleifera* (root, stem, leaf, flower, fruit capsule, and seed) were collected, dried, and ground into powder. Each tissue powder (1 g) was extracted with 10 mL of 70% ethanol under reflux for 2 h. The extract was filtered, concentrated, and then subjected to HPLC-ELSD analysis under the same conditions described above. The relative abundance of CSB1 in each tissue was compared based on peak intensity in HPLC-ELSD chromatograms.

The calibration curve was constructed using CSB1 reference standards at concentrations of 0.0417 mg/mL and 0.417 mg/mL. Due to the use of ELSD, a logarithmic linear model (lnA = 1.883·lnC + 15.858) was applied. The linear regression showed R^2^ > 0.999. The purity of CSB1 was determined to be >98% by peak area normalization (Supplementary Figure S1). The yield of CSB1 from camellia seed was approximately 9.34 mg per gram of dry material.

The extraction of CSB1 was performed following established methods. Powdered camellia seed cake was reflux-extracted with 70% ethanol, followed by sequential solvent partitioning. Further purification was achieved by silica gel column chromatography and reversed-phase C18 medium-pressure liquid chromatography followed by preparative HPLC to obtain high-purity CSB1 as a white powder [24].

### 2.3. Cell Culture

The cells were grown in DMEM supplemented with 10% fetal bovine serum along with a 1% Penicillin–Streptomycin mixture for incubation, at 37 °C in a humidified atmosphere containing 5% CO_2_. The culture medium was refreshed every 48 h. Upon reaching 80–90% confluence, the cells were passaged using 0.05% trypsin–EDTA. PC12 cells at passages 3–5 were used in all experiments. PC12 cells were divided into five groups: (1) Control group; (2) H_2_O_2_ group (500 µM H_2_O_2_); (3) H_2_O_2_ + CSB1 low-dose group (0.01 μM); (4) H_2_O_2_ + CSB1 medium-dose group (1 μM); and (5) H_2_O_2_ + CSB1 high-dose group (2 μM). CSB1 was dissolved in DMSO, with a final DMSO concentration of ≤0.1% (*v*/*v*) in the culture medium. For pretreatment, cells were incubated with CSB1 for 24 h. The CSB1-containing medium was then removed, and cells were washed twice with PBS and then incubated with fresh medium containing 500 µM H_2_O_2_ for 3 h. A vehicle control (0.1% DMSO) was included and showed no significant effect on cell viability (Figure S1). The concentration range (0.01–2 µM) was selected based on preliminary dose–response experiments (Figure S1), where 0.01 µM showed minimal protection, 1 µM showed moderate protection, and 2 µM showed maximal protection without cytotoxicity.

### 2.4. Cellular Survival Assessment

Cell viability was determined using a CCK-8 kit. PC12 cells (2 × 10^4^ cells/well) were cultured in 96-well plates. After the indicated treatments, the culture medium was removed, cells were washed once with PBS, and then 100 µL of fresh DMEM containing 10 µL of CCK-8 solution was added to each well. After incubation at 37 °C for 1 h, the absorbance at 450 nm was measured using a microplate reader.

### 2.5. Intracellular ROS Measurement

Intracellular ROS production was measured by utilizing the redox-responsive fluorescent dye DCFH-DA [25], according to the manufacturer’s instructions. A confocal microscope was utilized to detect the fluorescence signals emitted by 2,7-dichlorofluorescein (DCF) and capture corresponding images (Ex/Em = 504/529 nm).

### 2.6. CAT, GSH-Px, SOD and MDA Measurement

The activities of CAT, GSH-Px, SOD and MDA in PC12 cells were measured using biochemical assay kits (Jiancheng Biochemical, Nanjing, China) strictly according to the manufacturer’s instructions.

### 2.7. Scanning Electron Microscopy (SEM) for Cell Surface Morphology

Cells were collected according to the grouping and treatment methods described in previous sections. The culture medium was first removed, and the cells were immediately fixed with pre-warmed (to culture temperature) 2.5% glutaraldehyde at room temperature for 30 min. After three washes with 0.1 M phosphate buffer (5 min each), the cells were post-fixed with 1% osmium tetroxide at room temperature for 1 h to enhance membrane contrast and conductivity, followed by three washes with distilled water. Optionally, the cells were incubated in 1% carbohydrazide for 15–30 min, washed with distilled water, treated again with 1% osmium tetroxide for 30 min, and thoroughly rinsed with distilled water. The bottom of the culture dish was then cut into small pieces, dehydrated through a graded ethanol series (25%, 50%, 75%, and 100%) for 10 min each, and subjected to critical point drying using liquid CO_2_. The dried samples were mounted onto aluminum stubs with conductive adhesive, sputter-coated with a thin layer (5–10 nm) of gold, and finally examined under a scanning electron microscope to observe cell surface morphology. Observations were performed using a Hitachi SU8010 SEM (Hitachi High-Tech Corporation, Tokyo, Japan) at 15 kV.

### 2.8. Transmission Electron Microscopy (TEM) for Mitochondrial Ultrastructure

PC12 Cell samples were first fixed with pre-chilled 2.5% glutaraldehyde at 4 °C for at least 2 h, followed by three washes with 0.1 M sodium cacodylate buffer (pH 7.4) or PBS. Samples were then post-fixed with 1% osmium tetroxide (OsO_4_) at room temperature for 1–2 h to enhance membrane contrast, washed again, and dehydrated through a graded ethanol series (50%, 70%, 90%, and 100%) and propylene oxide. After dehydration, specimens were infiltrated and embedded in EPON resin, which was polymerized at 37 °C, 45 °C, and finally 60 °C. Ultrathin sections (70–80 nm) were cut using an ultramicrotome, collected on copper grids, and double-stained with 2% uranyl acetate followed by lead citrate to improve electron density. TEM observations were performed using a Hitachi HT7800 transmission electron microscope (Hitachi High-Tech Corporation, Tokyo, Japan) operated at 80 kV [26].

### 2.9. Mitochondrial Membrane Potential (MMP) Measurement

Following the respective drug treatments, PC12 cells from each group were incubated with the mitochondrial membrane potential indicator JC-1 at a final concentration of 5 μmol/L for 30 min at 37 °C. Cells from the control, H_2_O_2_, and CSB1 protection groups were then collected. According to the manufacturer’s instructions of the JC-1 assay kit (Beyotime Biotechnology, Shanghai, China), mitochondrial membrane potential was assessed using a flow cytometer with an excitation wavelength of 488 nm and emission wavelengths of 525 ± 20 nm (FL1 channel, monomeric form) and 585 ± 21 nm (FL2 channel, aggregate form). Data were analyzed using FlowJo^TM^ software V11 [27].

### 2.10. Intracellular Ca^2+^ Measurement

Intracellular mitochondrial calcium levels were measured using Rhod-2 AM (Beyotime Biotechnology, Shanghai, China), a fluorescent probe that preferentially accumulates in mitochondria. PC12 cells were seeded in 96-well cell culture plates and allowed to adhere before drug treatment. After treatment, PC12 cells were incubated with 5 µM Rhod-2 AM in HBSS at 37 °C for 30 min in the dark. Cells were then washed twice with PBS, and fluorescence signals were detected using a laser confocal microscope (Carl Zeiss AG, Oberkochen, Germany) with excitation at 552 nm and emission at 581 nm.

### 2.11. Oxygen Consumption Rate (OCR) Measurement

The OCR of intact cells was measured using the Mito Stress Test Kit and analyzed with a Seahorse XF96 Extracellular Flux Analyzer [28]. The Mito Stress Test Kit was purchased from Agilent Technologies (Santa Clara, CA, USA; catalog number 103015-100). PC12 cells were seeded in XF96 cell culture microplates and cultured at 37 °C. Cells from the control, H_2_O_2_, and CSB1 protection groups were then treated. Simultaneously, the calibration plate (containing calibration solution) and the assay compound mixture (OCR assay components: 1 µM oligomycin, 1 µM FCCP, 1 µM rotenone, and 1 µM antimycin A) were pre-incubated at 37 °C. The cell culture medium was then replaced with the assay solution, and OCR measurements were performed following the manufacturer’s protocol using the Agilent Seahorse XFe96 Analyzer (Agilent Technologies, Santa Clara, CA, USA).

### 2.12. Quantitative Real-Time PCR (qRT-PCR) Analysis

After treatment according to the experimental groups, total RNA was extracted using a commercial RNA extraction kit (Suzhou Xinsaimei Biotechnology, Suzhou, China). The purity and concentration of the extracted RNA were measured with a microspectrophotometer. Based on the RNA concentration, the appropriate volume for cDNA synthesis was calculated. The 20 µL reverse transcription reaction system was prepared in a 200 µL PCR tube by adding the corresponding reagents according to the manufacturer’s instructions of the reverse transcription kit. The resulting cDNA was stored at −20 °C for subsequent PCR amplification. Quantitative real-time PCR was performed using β-actin as the reference gene. Gene expression levels were evaluated using the 2^−ΔΔCt^ method. The following genes were analyzed: BCL2, BAX, BNIP3, NIX, LC3B, and β-actin (internal control). Primer sequences are listed in Table S1.

### 2.13. Western Blotting Analysis

After treatment according to the experimental grouping protocol, the cells were lysed with 1 mL of ice-cold lysis buffer containing protease inhibitors (PMSF). The lysates were centrifuged at 13,000× *g* for 10 min at 4 °C, and the supernatant was collected to obtain total protein. Protein concentration was determined using a BCA protein assay kit (Beyotime Biotechnology, Shanghai, China) Equal amounts of protein (20 µg per lane) were separated by 10% sodium dodecyl sulfate–polyacrylamide gel electrophoresis (SDS-PAGE) and transferred to a polyvinylidene difluoride (PVDF) membrane. The membrane was blocked with Beyotime QuickBlock™ blocking buffer for 1 h at room temperature, followed by incubation with primary antibodies against NIX (1:1000), BNIP3 (1:5000), LC3B (1:2500), BCL-2 (1:2000), BAX (1:2000), and β-actin (1:10,000) in TBST at 4 °C overnight. After washing, the membrane was incubated with corresponding horseradish peroxidase (HRP)-conjugated secondary antibodies (HRP at 1:100,000 dilution) for 1 h at room temperature. Protein bands were visualized using the Omni-ECL™ Basic Chemiluminescence Detection Kit Shanghai Epizyme Biomedical Technology, Shanghai, China).

### 2.14. Molecular Docking Simulation

The three-dimensional structures of the target proteins were retrieved from the Protein Data Bank (PDB). Specifically, the crystal structures of BCL2, BAX, BNIP3, NIX and LC3B were obtained. The three-dimensional structure of CSB1 was obtained from the PubChem database.

Receptor and ligand files were prepared using AutoDockTools v1.5.7. Protein preparation included the removal of water molecules, addition of polar hydrogens, assignment of Gasteiger charges, and conversion to PDBQT format. For the ligand (CSB1), all rotatable bonds were defined as flexible, and Gasteiger charges were assigned. A grid box of 60 × 60 × 60 Å was defined for each protein, centered on the predicted binding pocket based on the geometric center of the protein structure. Docking calculations were performed using AutoDock Vina v1.1.2 with an exhaustiveness value of 16. For each protein–ligand pair, 10 binding poses were generated and ranked according to the Vina binding energy score (kcal/mol). The representative pose with the lowest binding energy was selected for interaction analysis.

To validate the docking protocol, redocking of the co-crystallized ligand (where available) was performed, and the root-mean-square deviation (RMSD) between the predicted and experimental binding poses was calculated. Protein–ligand interactions were visualized and analyzed using PyMOL v2.5 and Discovery Studio v2021. Two-dimensional interaction diagrams were generated to illustrate hydrogen bonds, hydrophobic interactions, and other non-covalent contacts. It should be noted that these computational docking results are predictive and require experimental validation through methods such as surface plasmon resonance (SPR) or cellular thermal shift assays (CETSA) to confirm direct protein–ligand interactions.

### 2.15. Cell Immunofluorescence

PC12 cells were seeded into chamber slides and treated with indicated agents. The cells were washed with PBS 3 times and fixed with 4% paraformaldehyde for 15 min, washed 3 times, and permeabilized with TBST for 30 min. After blocked with 10% goat serum for 30 min, the cells were incubated with primary antibody (1:100) overnight at 4 °C. The cells were then incubated with Alexa Fluor 488 dye-conjugated secondary antibody (1:150) at 37 °C for 1 h in the dark. After washing three times with PBS, the cells were counterstained with Hoechst 33342 to stain the nuclei. PC12 cells were subsequently examined with an inverted fluorescence microscope.

### 2.16. Statistical Analysis

All experimental data are expressed as the mean ± standard deviation (SD) from at least three independent biological replicates. Normality was assessed using the Shapiro–Wilk test, and homogeneity of variances was evaluated using Levene’s test. Differences among multiple groups were analyzed using one-way analysis of variance (ANOVA) followed by Tukey’s post hoc test for multiple comparisons. A value of *p* < 0.05 was considered statistically significant. Differential expression analysis of transcriptomic data was performed using DESeq2 (version 1.38.3) within the R environment (version 4.2.1). Genes with |log_2_FC| ≥ 1 and an FDR-adjusted *p* < 0.05 (Benjamini–Hochberg correction) were considered differentially expressed. For all experiments, three independent biological replicates (*n* = 3) were performed unless otherwise specified. The cell viability assay (CCK-8) and enzyme activity assays were conducted with five independent biological replicates (n = 5), as indicated in the figure legends. The number of replicates did not vary among experimental groups within each assay. All graphs were generated using GraphPad Prism version 9.5.3. The transcriptomic data generated in this study have been deposited in the NCBI BioProject database under accession number PRJNA1390781 accessed on 15 May 2026 (https://www.ncbi.nlm.nih.gov/bioproject/PRJNA1390781).

## 3. Results

### 3.1. Identification of C. oleifera Seeds as a Rich Source of CSB1

**Figure 1:**
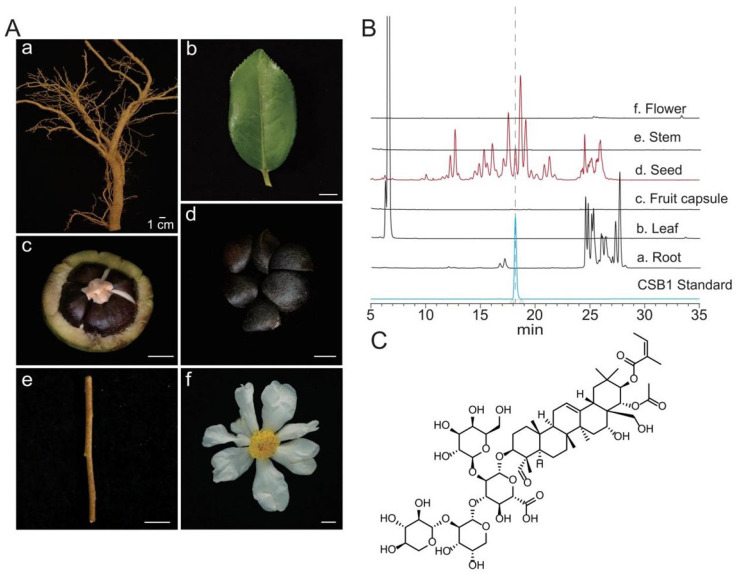
Tissue-specific distribution of CSB1 in *C. oleifera*. (**A**) Schematic diagram of tissue sampling from *C. oleifera*. (**a**) Root; (**b**) Leaf; (**c**) Fruit capsule; (**d**) Seed; (**e**) Stem; (**f**) Flower. (**B**) HPLC-ELSD chromatograms showing CSB1 peaks in extracts from different tissues. The characteristic peak of CSB1 is indicated by dashed line. (**C**) Chemical structure of CSB1.

To identify a natural source for the bioactive saponin CSB1, we first quantified its content in six different tissues of *C. oleifera*. As shown in Figure 1, among the six different tissues of *C. oleifera* (root, stem, leaf, flower, fruit capsule, and seed), the seed exhibited the highest CSB1 content, as indicated by the peak intensity in HPLC-ELSD chromatograms, with a content of 9.34 mg/g. Based on this finding, CSB1 was subsequently isolated and purified from the seeds of *C. oleifera* to investigate its cytoprotective potential in PC12 cells.

### 3.2. CSB1 Protects Against H_2_O_2_-Induced Oxidative Injury in PC12 Cells

**Figure 2:**
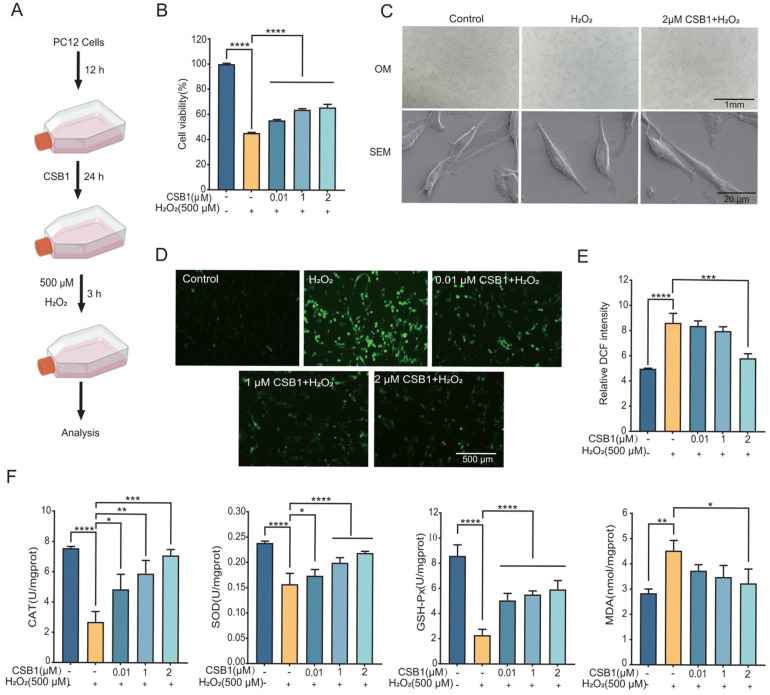
CSB1 increased antioxidant capacity and alleviated oxidative stress in PC12 cells. (**A**) Schematic Illustration of Drug Administration to PC12 cells. (**B**) The cell viability of PC12 Cells in different treatment groups. (**C**) CSB1 for H_2_O_2_ induced PC12 cell morphology (OM) and SEM visualization of cellular ultrastructure (SEM). (**D**) Fluorescence staining images of ROS in PC12 cells. (**E**) Statistics of DCF fluorescence intensity. (**F**) The levels of CAT, GSH-Px, SOD, MDA in different treatment groups. Data are presented as mean ± SEM (*n* = 5). Statistical significance was assessed using one-way ANOVA followed by Tukey’s post hoc test (* *p* < 0.05, ** *p* < 0.01, *** *p* < 0.001, **** *p* < 0.0001).

To evaluate the protective effect of CSB1, we established an in vitro model of H_2_O_2_-induced oxidative stress in PC12 cells. In the experimental setup, CSB1 was removed before H_2_O_2_ exposure; cells were pretreated with CSB1 for 24 h, followed by removal of CSB1 and subsequent treatment with fresh culture medium containing 500 μM H_2_O_2_ for 3 h. Initial experiments determined that a 3 h treatment with 500 µM H_2_O_2_ reduced cell viability to 45.29% (Figure S2), and this condition was selected for subsequent studies. PC12 cells were pretreated with 0.01, 1, or 2 μM CSB1 for 24 h prior to H_2_O_2_ exposure (Figure 2A). CSB1 pretreatment significantly mitigated the H_2_O_2_-induced loss of cell viability in a dose-dependent manner (Figure 2B). Morphological assessment by inverted and transmission electron microscopy demonstrated that H_2_O_2_ exposure caused cell rounding, shrinkage, loss of cellular protrusions, and a reduction in cell number. These morphological alterations were markedly prevented by CSB1 pretreatment (Figure 2C).

Additionally, CSB1 pretreatment significantly reduced the robust generation of intracellular ROS triggered by H_2_O_2_, as evidenced by a significant reduction in DCF fluorescence intensity (Figure 2D,E). CSB1 also dose-dependently enhanced the cellular antioxidant capacity in H_2_O_2_-injured cells, significantly elevating the activities of the antioxidant enzymes SOD, CAT, and GSH-Px while reducing the elevated content of the lipid peroxidation product MDA (Figure 2F). Collectively, these data demonstrate that CSB1 directly attenuates oxidative damage in PC12 cells by enhancing their antioxidant capacity.

### 3.3. CSB1 Exerts Cytoprotective Effects Through Regulation of the BNIP3/NIX-Mediated Mitophagy Pathway

**Figure 3:**
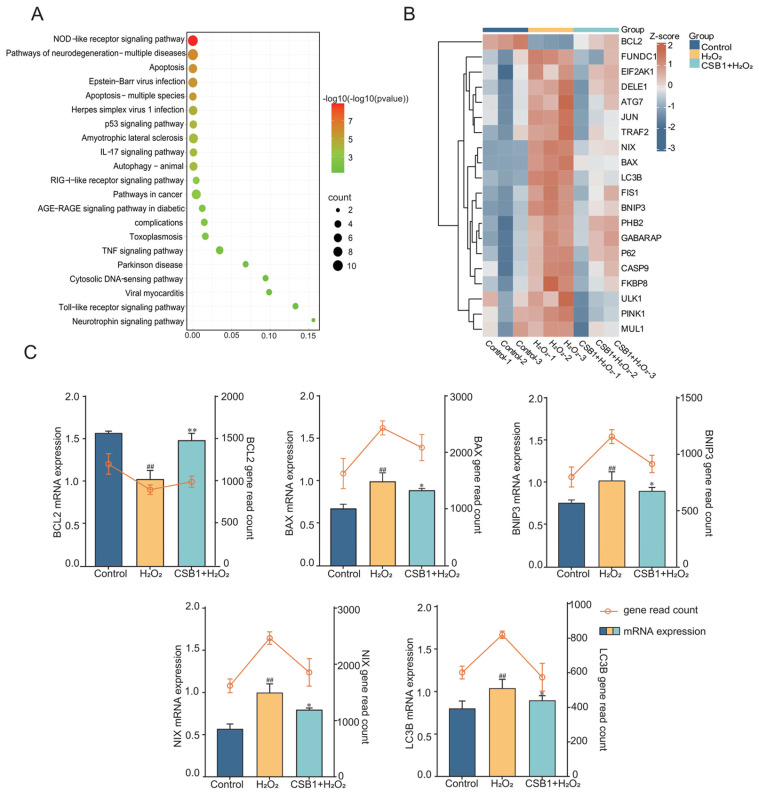
Transcriptome analysis of signaling pathways by which CSB1 affects PC12 cells. CSB1 was used at 2 µM (high dose) in these experiments unless otherwise specified. (**A**) KEGG pathway enrichment analysis of differentially expressed genes between the H_2_O_2_ and CSB1 + H_2_O_2_ groups. (**B**) Heatmap analysis of the RNA expression levels related to the mitophagy pathway. (**C**) Quantitative analysis of BCL2, BAX, BNIP3, NIX, and LC3B mRNA expression by qRT-PCR. Data are presented as mean ± SEM (*n* = 3). Statistical significance was assessed using one-way ANOVA followed by Tukey’s post hoc test (## *p* < 0.01 vs. Control group; * *p* < 0.05 vs. H_2_O_2_ group; ** *p* < 0.01 vs. H_2_O_2_ group).

To uncover the molecular basis of CSB1’s cytoprotection, we conducted transcriptome profiling. KEGG pathway analysis identified several significantly enriched pathways, including the NOD-like receptor, apoptosis, autophagy, and p53 signaling pathways (Figure 3A). We therefore hypothesized that CSB1 mitigates injury by modulating autophagic processes. Transcriptomic and subsequent qRT-PCR validation consistently demonstrated that H_2_O_2_ injury induces a dysregulated autophagy/apoptosis program, characterized by the upregulation of BAX, BNIP3, NIX, and LC3B and the downregulation of BCL2. CSB1 pretreatment effectively normalized the expression of these key mediators (Figure 3B) and was further verified by qRT-PCR (Figure 3C). Collectively, these findings establish that CSB1 protects against H_2_O_2_-induced damage by rectifying the imbalance in the autophagy signaling pathway. CSB1 restores mitochondrial homeostasis and curbs excessive mitophagy and apoptosis primarily by upregulating BCL2 and suppressing pro-apoptotic/autophagic genes such as BAX and BNIP3.

### 3.4. CSB1 Attenuates H_2_O_2_-Induced Apoptosis and Ameliorates MMP in PC12 Cells

**Figure 4:**
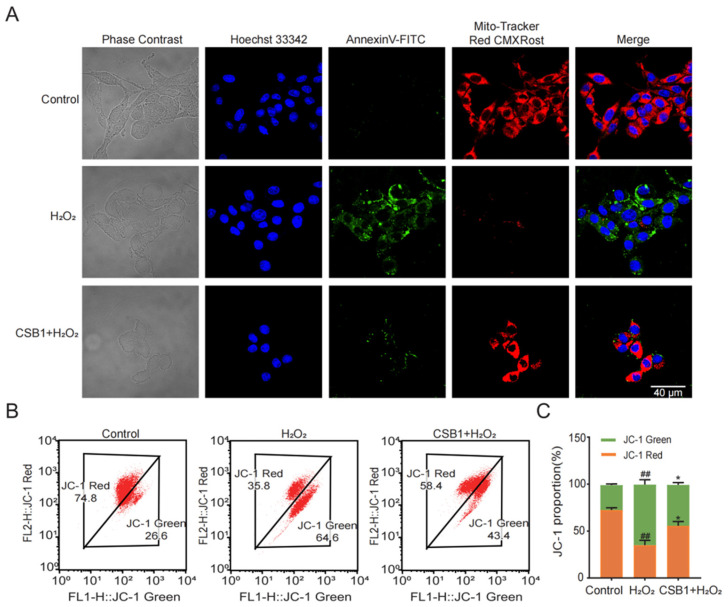
CSB1 attenuates H_2_O_2_-induced apoptosis and ameliorates MMP (ΔΨm) dissipation in PC12 cells. CSB1 was used at 2 µM (high dose) in these experiments unless otherwise specified. (**A**) Representative fluorescent images showing the protective effects of CSB1 on H_2_O_2_-induced apoptosis and ΔΨm loss. (Hoechst 33342 stains the nuclei of live cells, Annexin V-FITC labels apoptotic cells, and MitoTracker Red CMXRos indicates mitochondrial membrane potential.) (**B**) Analysis of ΔΨm by flow cytometry using JC-1 staining. (**C**) JC-1 proportion of mitochondrial membrane potential. Data are presented as mean ± SEM (*n* = 3). Statistical significance was assessed using one-way ANOVA followed by Tukey’s post hoc test (## *p* < 0.01 vs. Control group; * *p* < 0.05 vs. H_2_O_2_ group).

The protective effect of CSB1 against H_2_O_2_-induced apoptosis and mitochondrial impairment was assessed. As quantified in Figure 4A, H_2_O_2_ exposure significantly increased the apoptosis rate, an effect that was dose-dependently attenuated by CSB1 pretreatment. Fluorescence microscopy revealed correlative morphological and functional changes: control cells displayed normal morphology, Annexin V-FITC negativity, and strong Mito-Tracker Red fluorescence, indicating healthy MMP (ΔΨm). In contrast, H_2_O_2_-treated cells exhibited apoptotic morphology, positive Annexin V-FITC staining, and markedly diminished Mito-Tracker Red fluorescence, indicating severe mitochondrial depolarization. In contrast, the CSB1 pretreatment effectively preserved both normal cellular architecture and Mito-Tracker Red fluorescence intensity.

Furthermore, the MMP was quantitatively analyzed using the JC-1 probe via flow cytometry (Figure 4B,C). Consistent with the microscopic findings, H_2_O_2_ treatment significantly reduced the red/green fluorescence ratio (JC-1 aggregates/monomers) compared to the control (*p* < 0.001), indicating MMP collapse. CSB1 pretreatment significantly and dose-dependently restored this ratio, confirming the preservation of MMP. In summary, CSB1 counteracts H_2_O_2_-induced cytotoxicity by suppressing apoptosis and preserving MMP.

### 3.5. CSB1 Attenuates H_2_O_2_-Induced Cell Injury by Preserving Mitochondrial Function

**Figure 5:**
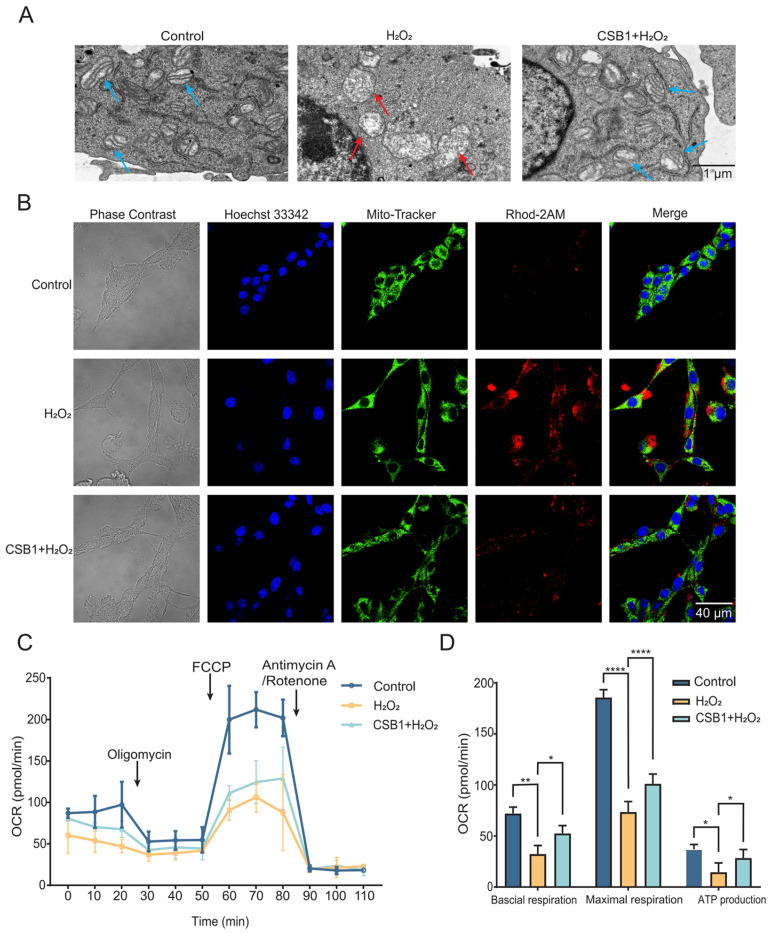
CSB1 mitigates H_2_O_2_-induced cellular damage by preserving mitochondrial bioenergetic function in PC12 cells. CSB1 was used at 2 µM (high dose) in these experiments unless otherwise specified. (**A**) Representative transmission electron microscopy (TEM) images of mitochondrial ultrastructure. Red arrows indicate representative damaged mitochondria with swelling, cristae disruption, and vacuolization; blue arrows indicate normal or protected mitochondria. (**B**) Assessment of mitochondrial calcium levels using the fluorescent probe Rhod-2 AM. Red fluorescence indicates mitochondrial calcium accumulation. (**C**) Quantitative assessment of mitochondrial respiratory function measurement. (D) OCR of mitochondrial function. Data are presented as mean ± SEM (*n* = 3). Statistical significance was assessed using one-way ANOVA followed by Tukey’s post hoc test (* *p* < 0.05, ** *p* < 0.01, **** *p* < 0.0001).

To delineate the cytoprotective mechanisms of CSB1, we systematically evaluated its impact on mitochondrial ultrastructure, calcium homeostasis, and respiratory function following H_2_O_2_ insult. First, transmission electron microscopy demonstrated that CSB1 effectively preserved mitochondrial structural integrity against H_2_O_2_-induced damage, preventing swelling, cristae disruption, and vacuolization (Figure 5A). As shown in Figure 5B, H_2_O_2_ exposure markedly increased mitochondrial calcium levels (indicated by enhanced Rhod-2 AM fluorescence), while CSB1 pretreatment significantly attenuated this increase. Finally, mitochondrial respiratory function was quantitatively evaluated by measuring the oxygen consumption rate (OCR). Functional analysis revealed that CSB1 rescued the impaired oxidative phosphorylation capacity caused by H_2_O_2_. Specifically, it restored basal and maximal respiration and ATP production while significantly enhancing maximal respiratory capacity (Figure 5C,D). Thus, CSB1 exerts multi-faceted protection on mitochondria by preserving their structure, maintaining calcium homeostasis, and augmenting bioenergetic function, which collectively underpin its anti-apoptotic effect.

### 3.6. CSB1 Reduces BNIP3/NIX-LC3B-Related Responses Under Oxidative Stress

**Figure 6:**
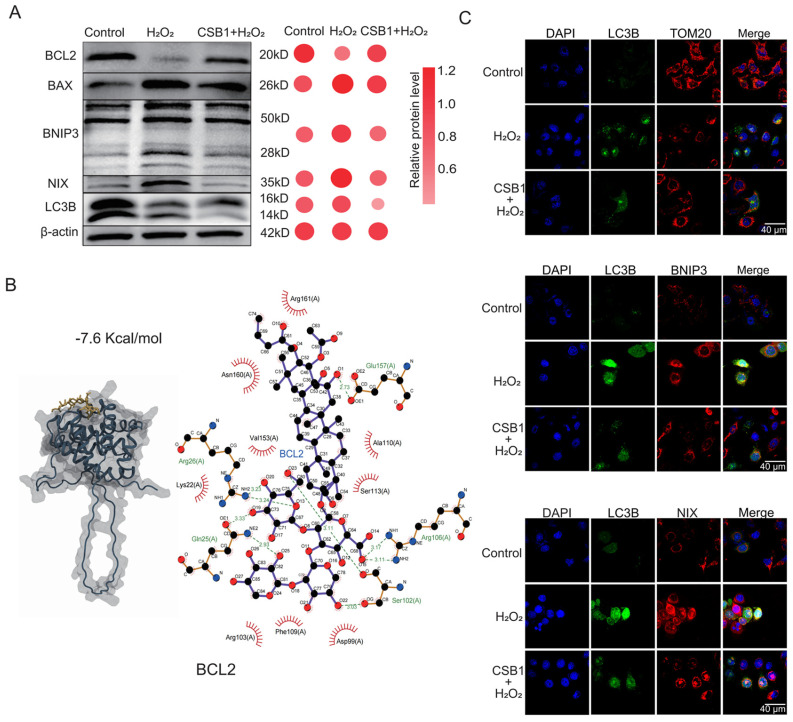
CSB1 interacts with mitophagy proteins and regulates their expression. CSB1 was used at 2 µM (high dose) in these experiments unless otherwise specified. (**A**) Protein levels of apoptosis-related markers (BAX, BCL2) and mitophagy-related markers (BNIP3, NIX, LC3B) were detected by Western blot. (**B**) Predicted binding modes and interaction patterns of CSB1 with BCL2, BAX, BNIP3, NIX, and LC3B. Binding energies (kcal/mol): BCL2: −7.6, BAX: −6.9, BNIP3: −5.2, NIX: −5.0, LC3B: −7.7. Protein structures are shown in gray, and the ligand (CSB1) is shown in yellow. (Additional results are provided in Supplementary Figure S4.) (**C**) Representative immunofluorescence images showing the subcellular localization and expression levels of LC3B, BNIP3, and NIX. Nuclear were stained with DAPI (blue).

To validate the preceding findings at the protein level and further examine BNIP3/NIX-LC3B-related responses, we performed Western blotting, molecular docking, and immunofluorescence experiments. Western blot analysis (Figure 6A) confirmed the transcriptomic results, showing that H_2_O_2_ injury significantly upregulated the protein levels of BAX, BNIP3, NIX, and LC3B-II while downregulating BCL2. Molecular docking was performed using AutoDock Vina v1.1.2 to predict potential interactions between CSB1 and mitophagy/apoptosis-related proteins (BCL2, BAX, BNIP3, NIX, LC3B). As summarized in Figure 6B and Supplementary Figure S4, the predicted binding energies ranged from −5.0 to −7.7 kcal/mol across all tested proteins. The lowest predicted binding energies were obtained for LC3B (−7.7 kcal/mol) and BCL2 (−7.6 kcal/mol), followed by BAX (−6.9 kcal/mol), BNIP3 (−5.2 kcal/mol), and NIX (−5.0 kcal/mol). Two-dimensional interaction maps of BCL2 revealed specific residue–ligand contact patterns. CSB1 formed multiple polar contacts with surrounding residues, including Glu157, Arg26, Arg106, Gln25, and Ser102. Additional nearby residues, such as Arg161, Asn160, Val153, Ala110, Lys22, Ser113, Arg103, Asp99, and Phe109, were positioned near the ligand and may contribute to stabilizing the binding conformation (Figure 6B). Similar interaction analyses for BAX, BNIP3, NIX, and LC3B are provided in Supplementary Figure S4. Immunofluorescence co-localization assays (Figure 6C) functionally validated these interactions, showing significantly enhanced BNIP3/NIX-LC3B co-localization (yellow puncta) in H_2_O_2_-injured cells, indicating activated mitophagosome formation. CSB1 pretreatment reduced the expression of mitophagy-related proteins (BNIP3, NIX, LC3B-II) and BNIP3/NIX-LC3B co-localization.

## 4. Discussion

**Figure 7:**
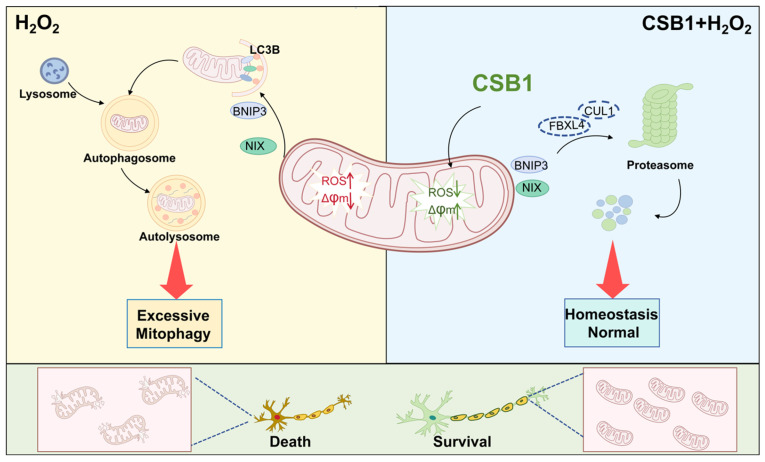
The mechanism by which camelliasaponin B1 inhibits BNIP3/NIX-mediated excessive mitophagy. PC12 cells, a rat adrenal pheochromocytoma cell line widely used as a neuronal model for studying oxidative stress and neuroprotection.

Our findings reveal that CSB1, a saponin derived from *C. oleifera* seed cake by-product, confers neuroprotection against H_2_O_2_-induced oxidative damage in PC12 cells through a previously unrecognized mechanism involving BNIP3/NIX-mediated mitophagy regulation. The cytoprotective effects are mediated by enhancing endogenous antioxidant defenses, preserving mitochondrial integrity, and reducing the expression of BNIP3/NIX-LC3B pathway-related proteins and their co-localization (Figure 7). Beyond the compound’s pharmacological interest, this work also provides a mechanistic rationale for valorizing this underutilized agricultural residue, aligning with emerging interests in the circular bioeconomy.

We first established that CSB1 significantly alleviates H_2_O_2_-induced cytotoxicity and oxidative stress. Oxidative stress, characterized by an imbalance between ROS production and antioxidant capacity, is a key contributor to neuronal damage in neurodegenerative disorders [29,30]. In our experimental model, H_2_O_2_ triggered a sharp increase in intracellular ROS, resulting in lipid peroxidation (as indicated by elevated MDA levels) and a marked decline in cell viability. Pretreatment with CSB1 not only restored cell viability in a dose-dependent manner and scavenged excess ROS but also enhanced the activities of major antioxidant enzymes, including SOD, CAT, and GSH-Px. These results suggest that the protective action of CSB1 extends beyond direct radical scavenging to include potent reinforcement of the endogenous antioxidant defense system. Notably, CSB1 is a chemically defined compound derived from a unique botanical source, which offers advantages over crude saponin mixtures or extracts from other species.

CSB1 exerts cytoprotection primarily by safeguarding mitochondrial homeostasis, identifying mitochondria as its key intracellular target. As both the primary source and major target of ROS, mitochondria constitute a central hub in oxidative stress-induced apoptosis [31,32]. Persistent oxidative injury triggers mitochondrial permeability transition pore opening, loss of ΔΨm, and activation of the intrinsic apoptotic pathway [33,34]. Our findings provide multi-level evidence that CSB1 pretreatment mitigates this mitochondrial injury cascade: it prevented ΔΨm dissipation, quantified by restored JC-1 aggregate/monomer ratios, thereby preserving the electrochemical gradient essential for ATP production. Ultrastructural analysis via TEM revealed that CSB1 mitigated H_2_O_2_-induced mitochondrial swelling, cristae disruption, and outer membrane rupture. CSB1 alleviated mitochondrial calcium overload, a key event precipitating necrotic and apoptotic signaling. Most significantly, Seahorse XF analysis demonstrated that CSB1 restored OXPHOS capacity, rescuing basal and maximal respiration along with ATP production, which is critical for meeting neuronal energy demands [35]. Together, this tripartite preservation of mitochondrial integrity, ion homeostasis, and bioenergetic function establishes mitochondrial protection as a central mechanism underlying CSB1’s anti-apoptotic efficacy.

Mechanistically, transcriptomic and qRT-PCR analyses revealed that H_2_O_2_ injury upregulates mitophagy receptors (BNIP3, NIX), autophagy marker LC3B, and pro-apoptotic BAX while downregulating anti-apoptotic BCL-2. CSB1 pretreatment reversed these changes, shifting the transcriptome toward survival. Notably, CSB1 also upregulated BCL-2, suggesting a dual mechanism: transcriptional suppression of pro-mitophagy genes and enhancement in anti-apoptotic signaling. Western blot analysis confirmed that CSB1 pretreatment significantly suppressed H_2_O_2_-induced upregulation of BNIP3, NIX, and LC3B-II while restoring BCL-2 levels. Functionally, immunofluorescence assays revealed that CSB1 substantially reduced the enhanced BNIP3/LC3B and NIX/LC3B co-localization following H_2_O_2_ injury, providing spatial evidence of inhibited mitophagosome formation. Molecular docking simulations further indicated that CSB1 may form stable, energetically favorable interactions with BNIP3, NIX, and LC3B (binding energies < −5 kcal/mol), suggesting possible protein–ligand interactions that require experimental validation. Unlike conventional antioxidants that broadly scavenge ROS, CSB1 acts upstream by suppressing the transcriptional and protein expression of BNIP3/NIX, thereby restoring mitophagic balance. This BNIP3/NIX-LC3B-associated response may contribute to its efficacy at low micromolar concentrations (0.01–2 μM) and highlights the potential of CSB1 as a chemical probe for dissecting BNIP3/NIX-dependent mitophagy pathways.

CSB1’s capacity to fine-tune mitophagy makes it a promising candidate for therapeutic strategies in neurodegenerative diseases, where mitochondrial quality control is fundamentally impaired. In conditions such as Parkinson’s and Alzheimer’s disease, mitophagy plays a dual role: essential for clearance of damaged mitochondria yet pathogenic when dysregulated [36,37]. CSB1 restores mitophagic balance, preventing excessive loss of functional mitochondria and subsequent bioenergetic collapse without completely abolishing this vital process. Mechanistically, CSB1 acts through the BNIP3/NIX pathway, which operates independently of the PINK1–Parkin axis and is associated with hypoxic/oxidative stress responses [22,38], suggesting broader applicability in mitochondrial pathologies beyond those involving Parkin mutations. Recent work reveals that BNIP3/NIX stability is regulated by the ubiquitin–proteasome system, including the SCF-FBXL4 E3 ligase complex and adaptor PPTC7 [39,40]. While our study did not explore this upstream layer, it raises the compelling possibility that CSB1 may influence BNIP3/NIX ubiquitination and turnover, opening a clear path for future investigation.

Several limitations of this study should be acknowledged. Direct binding assays (e.g., SPR, CETSA) for CSB1-BNIP3/NIX interactions were not performed; thus, whether CSB1 directly binds these targets remains to be validated. Mitophagic flux was not assessed using lysosomal inhibitors or mit-Keima; our conclusions are based on steady-state protein levels. TOM20 staining was not performed, limiting assessment of mitochondrial outer membrane integrity. CSB1-only controls for OCR and mitophagy markers were not included, though CSB1 alone showed no effect on cell viability, ROS, or antioxidant enzymes. Full structural characterization by NMR or LC-MS/MS was not conducted; CSB1 was identified by HPLC-ELSD and reference standard comparison. Finally, while indirect evidence suggests CSB1 enters cells, direct quantification of intracellular CSB1 was not performed. Future studies addressing these limitations are needed.

## 5. Conclusions

In conclusion, this study shows that CSB1 is associated with protective effects against oxidative stress in PC12 cells. Our findings suggest that CSB1 may modulate mitochondrial quality control through three coordinated processes: (1) enhancing endogenous antioxidant defenses; (2) preserving mitochondrial structural and functional integrity; and (3) reducing the expression of BNIP3/NIX-LC3B pathway-related proteins and their co-localization. These results position CSB1 as a promising lead compound for developing neuroprotective agents or functional food ingredients targeting oxidative stress-related mitochondrial dysfunction. Further studies are required to determine whether CSB1 directly binds to BNIP3/NIX or acts through indirect mechanisms.

## Data Availability

The original data presented in the study are openly available in NCBI BioProject (SRA) at https://www.ncbi.nlm.nih.gov/bioproject/PRJNA1390781.

## Supplementary Materials

The following supporting information can be downloaded at: https://www.mdpi.com/article/10.3390/antiox15070824/s1, Table S1: Primers used in qRT-PCR analyses; Figure S1. Representative HPLC-ELSD chromatogram of the CSB1 reference standard; Figure S2. Establishment of oxidative stress model in PC12 cells; Figure S3. Effects of CSB1 alone on baseline cellular parameters; Figure S4. Detailed molecular docking interactions between CSB1 and target proteins; Figure S5. Densitometric quantification of Western blot bands shown in Figure 6A.

## Abbreviations

The following abbreviations are used in this manuscript:

CSB1	Camelliasaponin B1
ROS	Reactive Oxygen Species
H_2_O_2_	Hydrogen Peroxide
CAT	Catalase
SOD	Superoxide Dismutase
MDA	Malondialdehyde
GSH-Px	Glutathione Peroxidase
BAX	BCL2-Associated X Protein
BCL2	B-Cell Lymphoma 2
BNIP3	BCL2 Interacting Protein 3
NIX	NIP3-like protein X (BNIP3L)
LC3B	Microtubule-Associated Protein 1 Light Chain 3 Beta
FUNDC1	FUN14 Domain Containing 1
PINK1	PTEN-Induced Putative Kinase 1
MPTP	Mitochondrial Permeability Transition Pore
PPTC7	PTC7 Protein Phosphatase Homolog
