# Supplementary material for: Camelliasaponin B1, a Saponin from Camellia oleifera Seed, Protects Against Oxidative Stress and Is Associated with Reduced BNIP3/NIX-LC3B Expression in PC12 Cells
Source: Antioxidants (Basel). 2026 Jun 30;15(7):824. doi: 10.3390/antiox15070824 (PMC13406071; doi:10.3390/antiox15070824)
Supplement: Supplementary file 1 [file antioxidants-15-00824-s001.zip › antioxidants-4336106-supplementary.pdf]

Camelliasaponin B1, a saponin from Camellia oleifera seed, protects against oxidative stress and is associated with reduced BNIP3/NIX-LC3B expression in PC12 cells

Table S1 | Primers used in qRT-PCR analyses.

S.NO.	Gene name	Accession Number	Primer sequences
1.	*Bcl2*-RT-F	NM_016993.2	GAGGGGCTACGAGTGGGATA
	Bcl2-RT-R		CGGTAGCGACGAGAGAAGTC
2.	*Bax*-RT-F	NM_017059.2	CAACATGGAGCTGCAGAGGA
	Bax-RT-R		GGAAAGGAGGCCATCCCAG
3.	*Bnip3*-RT-F	NM_053420.3	GATGCGCAGCATGAATCTGG
	Bnip3-RT-R		CTGAGAGTAGCTGTGCGCTT
4.	*Nix*-RT-F	NM_080888.3	TCTCACTTAGTCGAGCCGCC
	Nix-RT-R		TTTGCCCGTCTTCTTGTGGT
5.	*LC-3b*-RT-F	NM_022867.2	GAAGACCTTCAAACAGCGCC
	LC-3b-RT-R		CCAGGAGGAAGAAGGCTTGG
6.	*β-actin*-RT-F	NM_031144.3	AGGCTGTGTTGTCCCTGTATG
	β-actin-RT-R		AACCGCTCATTGCCGATAGT

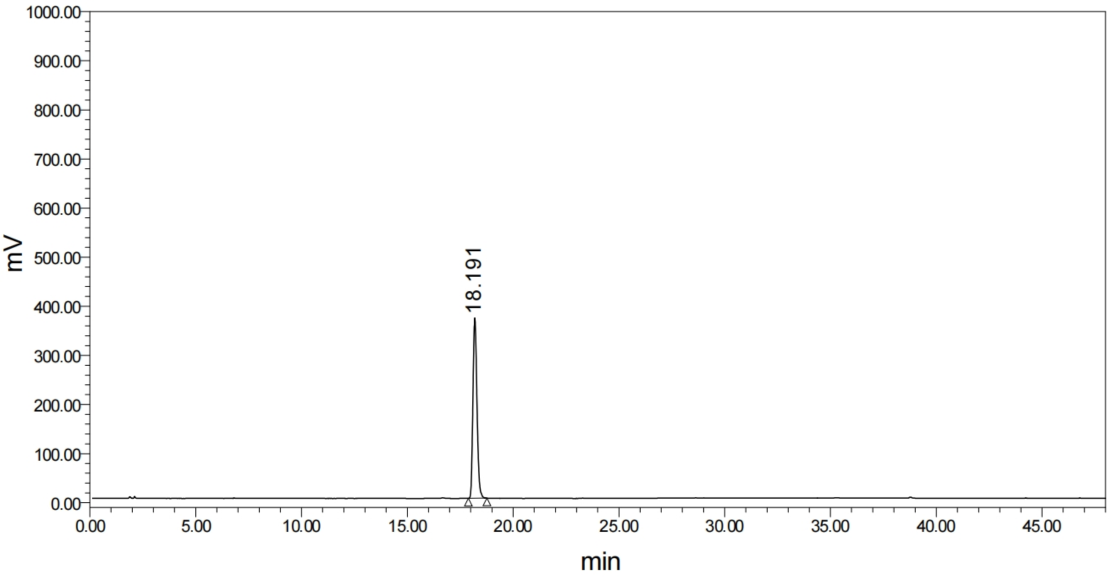

Figure S1. Representative HPLC- ELSD chromatogram of the CSB1 reference standard. The purity was determined to be >98% by peak area normalization.

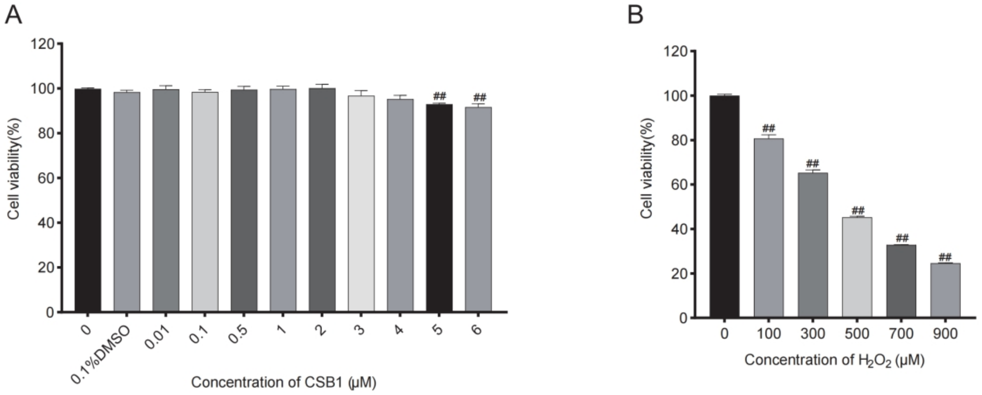

Figure S2. Establishment of oxidative stress model in PC12 cells. (A) The effect of incubation concentration of CSB1 on PC12 cell viability. (B) The effect of incubation concentration of H_2O_2 on PC12 cell viability.Data are presented as mean ± SEM (n=5,i.e.five in dependent replicates) $^{\#}p < 0.05$, $^{\#\#}p < 0.01$.

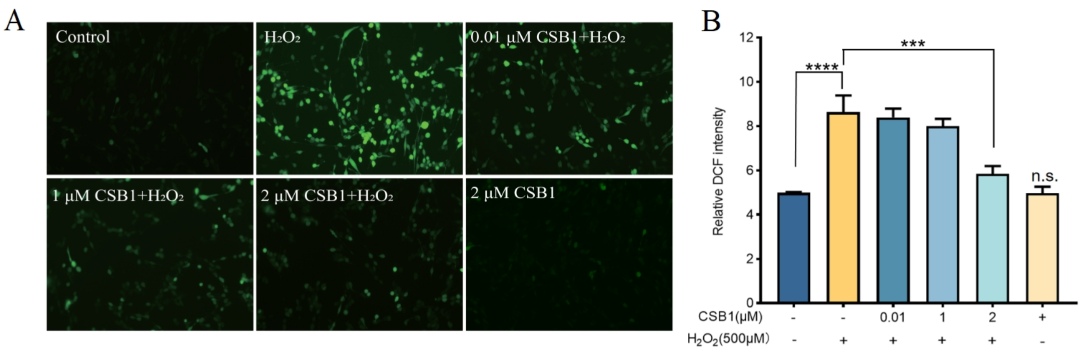

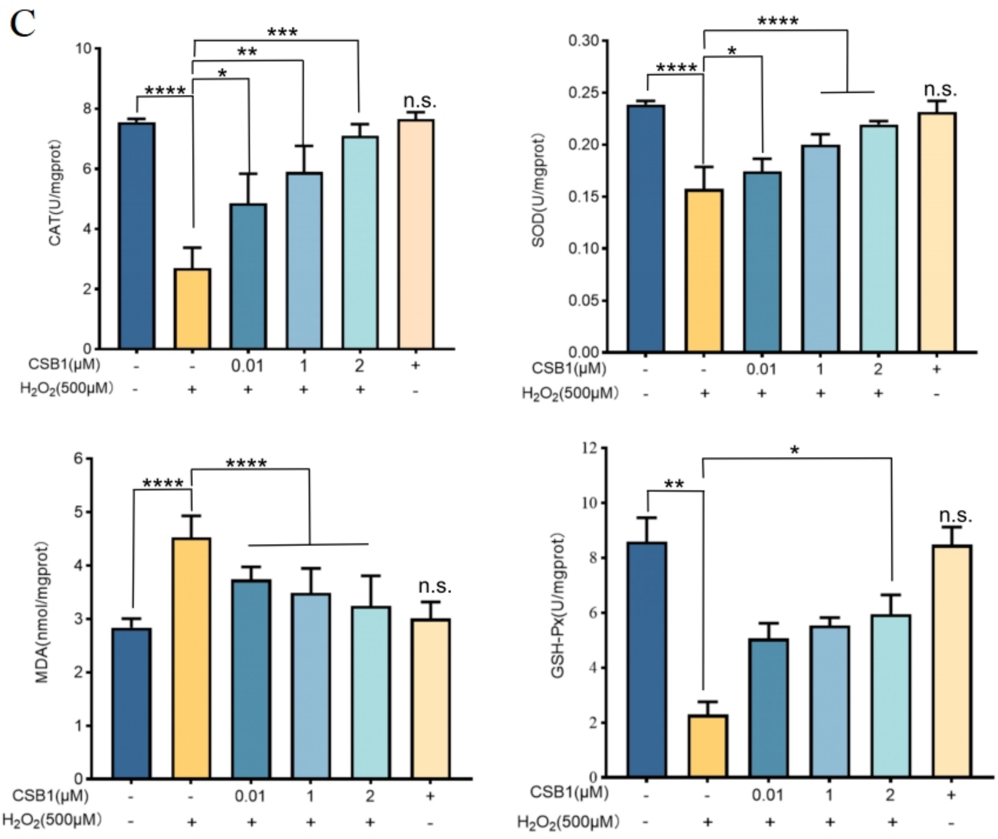

Figure S3. Effects of CSB1 alone on baseline cellular parameters. PC12 cells were treated with 2 μM CSB1 alone for 24 h. (A) Intracellular ROS levels. (B) Statistics of DCF fluorescence intensity. (C) Activities of SOD, CAT, GSH-Px and MDA content. Data are presented as mean ± SD (n = 5).n.s. indicates not significant vs. control group . *$p < 0.05$, **$p < 0.01$, ***$p < 0.001$, ****$p < 0.0001$.

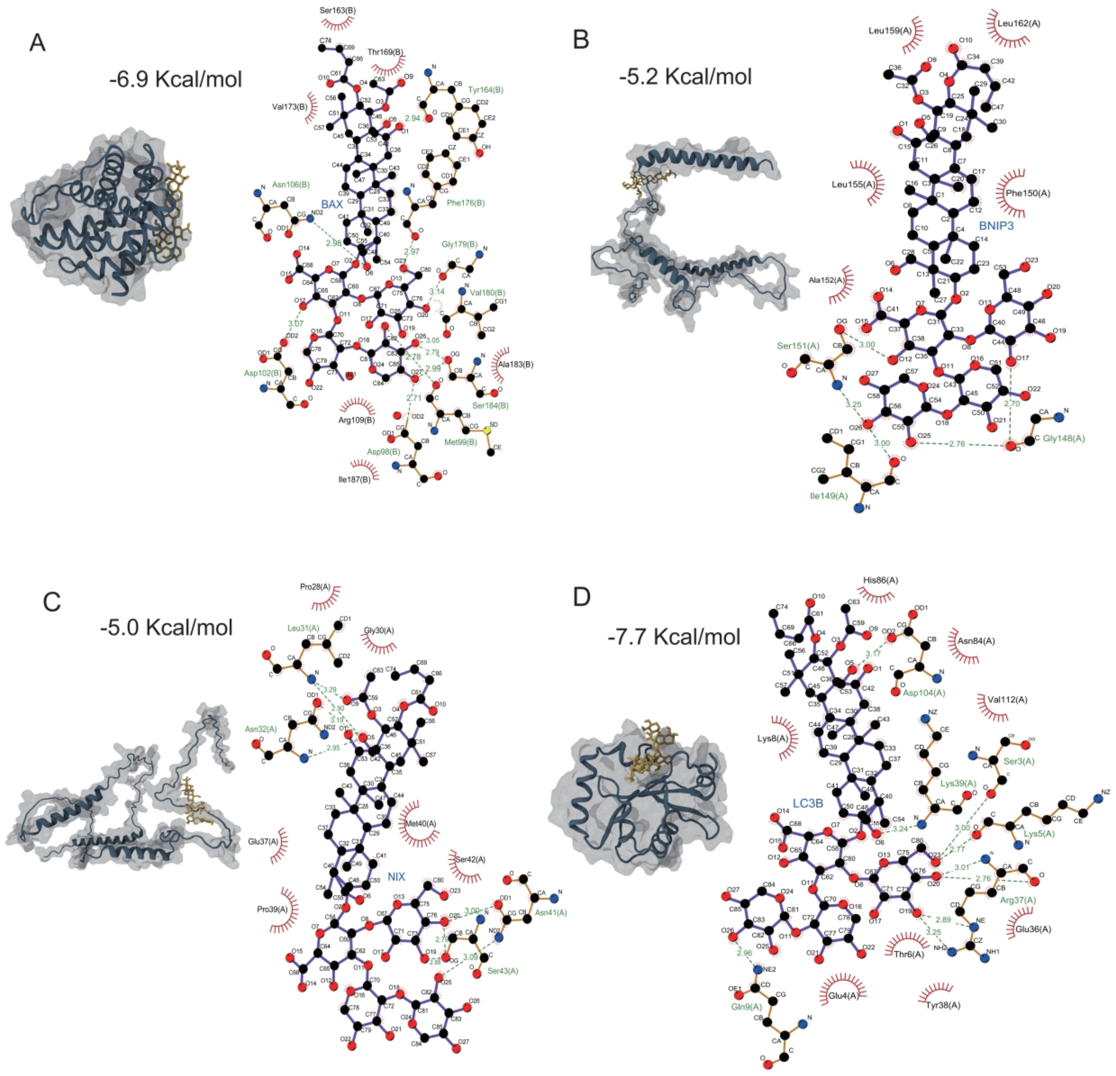

Figure S4. Detailed molecular docking interactions between CSB1 and target proteins. (A) BAX-CSB1complex; (B)BNIP3-CSB1complex; (C)NIX-CSB1complex; (D)LC3B-CSB1 complex. Hydrogen bonds are shown as yellow dashed lines, and hydrophobic interactions as gray dashed lines.Binding energies are indicated for each complex.

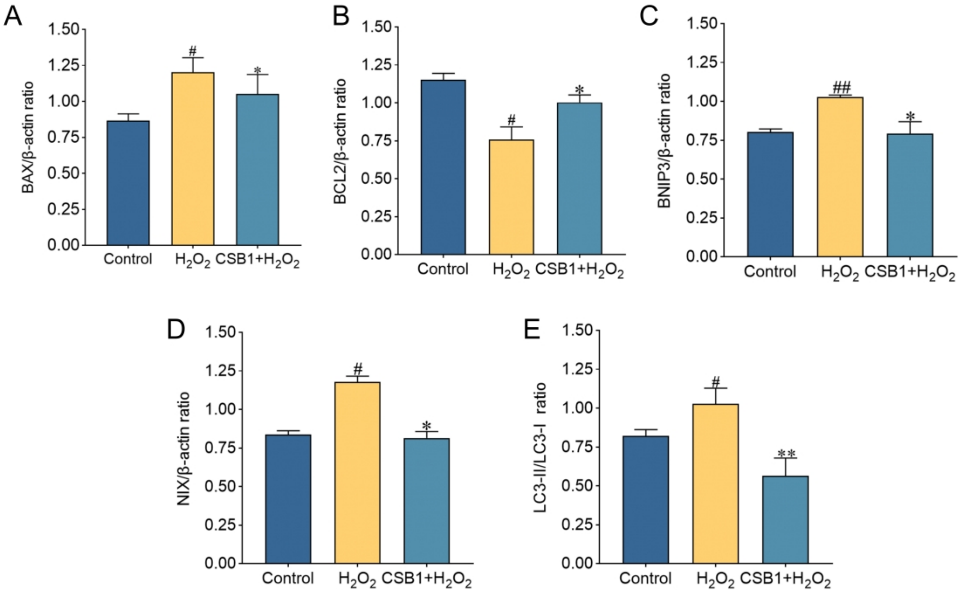

Figure S5. Densitometric quantification of Western blot bands shown in Figure 6A. Protein expression levels were normalized to β-actin. Data are presented as mean ± SD (n = 3 independent biological replicates). Statistical analysis: one-way ANOVA with Tukey's post hoc test.#p < 0.05 vs Control group ; ##p < 0.01 vs Control group; *p < 0.05 vs H_2O_2 group; **p < 0.01 vs H_2O_2 group.
